# PetCO_2_, VCO_2_ and CorPP Values in the Successful Prediction of the Return of Spontaneous Circulation: An Experimental Study on Unassisted Induced Cardiopulmonary Arrest

**DOI:** 10.5935/1678-9741.20160093

**Published:** 2016

**Authors:** Ana Carolina Longui Macedo, Luiz Claudio Martins, Ilma Aparecida Paschoal, Carlos Cesar Ivo Sant'Ana Ovalle, Sebastião Araújo, Marcos Mello Moreira

**Affiliations:** 1 Universidade Estadual de Campinas (UNICAMP), Campinas, SP, Brazil.; 2 Universidade Paulista, São Paulo, SP, Brazil.

**Keywords:** Heart Arrest, Induced, Cardiopulmonary Resuscitation, Capnography, Epinephrine

## Abstract

**Introduction:**

During cardiac arrest, end-tidal CO_2_ (PetCO_2_), VCO_2_ and coronary perfusion pressure fall abruptly and tend to return to normal levels after an effective return of spontaneous circulation. Therefore, the monitoring of PetCO_2_ and VCO_2_ by capnography is a useful tool during clinical management of cardiac arrest patients.

**Objective:**

To assess if PetCO_2_, VCO_2_ and coronary perfusion pressure are useful for the prediction of return of spontaneous circulation in an animal model of cardiac arrest/cardiopulmonary resuscitation treated with vasopressor agents.

**Methods:**

42 swine were mechanically ventilated (FiO_2_=0.21). Ventricular fibrillation was induced and, after 10 min, unassisted cardiac arrest was initiated, followed by compressions. After 2 min of basic cardiopulmonary resuscitation, each group received: Adrenaline, Saline-Placebo, Terlipressin or Terlipressin + Adrenaline. Two minutes later (4^th^ min of cardiopulmonary resuscitation), the animals were defibrillated and the ones that survived were observed for an additional 30 min period. The variables of interest were recorded at the baseline period, 10 min of ventricular fibrillation, 2^nd^ min of cardiopulmonary resuscitation, 4^th^ min of cardiopulmonary resuscitation, and 30 min after return of spontaneous circulation.

**Results:**

PetCO_2_ and VCO_2_ values, both recorded at 2 min and 4 min of cardiopulmonary resuscitation, have no correlation with the return of spontaneous circulation rates in any group. On the other hand, higher values of coronary perfusion pressure at the 4th min of cardiopulmonary resuscitation have been associated with increased return of spontaneous circulation rates in the adrenaline and adrenaline + terlipressin groups.

**Conclusion:**

Although higher values of coronary perfusion pressure at the 4th min of cardiopulmonary resuscitation have been associated with increased return of spontaneous circulation rates in the animals that received adrenaline or adrenaline + terlipressin, PetCO_2_ and VCO_2_ have not been shown to be useful for predicting return of spontaneous circulation rates in this porcine model.

**Table:** 

**Abbreviations, acronyms & symbols**	
ADR	=Adrenaline
AHA	=American Heart Association
CA	=Cardiac arrest
CorPP	=Coronary perfusion pressure
CPR	=Cardiopulmonary resuscitation
DC	=Decreased cardiac output
IMV	=Invasive mechanical ventilation
PetCO^2^	=End-tidal CO_2_
ROSC	=Return of spontaneous circulation
TP	=Terlipressin
VF	=Ventricular fibrillation
V/Q	=Ventilation/perfusion ratio

## INTRODUCTION

Cardiac arrests occur daily in large numbers in several countries of the world and, for the most part, result in death. Cardiopulmonary resuscitation (CPR) is proposed by the American Heart Association (AHA) as an easy intervention, with the goal of reducing the number of deaths, despite discouraging statistics showing that only a small number of patients survive after this event. The use of methods that assess the effectiveness of CPR and that are preferably non-invasive, indicating the metabolic state and the dynamics of the cardiovascular system, would be of great value. Essentially, CPR consists of manual compressions of the patient's thorax, in an effort to help create an artificial anterograde blood flow, combined with either a noninvasive ventilation technique (*e.g*., mouth-to-mouth) or invasive mechanical ventilation in order to oxygenate the blood that reaches the lungs^[^^1^^]^.

Capnography presents itself as a non-invasive method, applicable at the bedside, that allows for the assessment of cardiorespiratory status both in experimental^2^^-^^6^^]^ and clinical studies^[^^7^^-^^12^^]^. In addition, it is considered an indicator and/or guide for decisions that enables the assessment of the quality of the CPR maneuvers^[^^13^^]^. Capnography evaluates and monitors physiological conditions by measuring exhaled CO_2_ through an infrared light sensor. CO_2_ excretion (VCO_2_) and partial pressure of CO_2_ at the end of the exhalation (PetCO_2_) are indicative of O_2_ consumption by the oxidative metabolism in the tissues and those values are closely related to the pulmonary ventilation/perfusion ratio (V/Q); hence, it is expected that both will increase in an effective CPR. Therefore, the monitoring of exhaled CO_2_ has been proposed and used as a non-invasive method to assess cardiorespiratory function, especially in situations of decreased cardiac output (DC), such as during shock and CPR, and its use is compulsory in Surgical Centers^[^^14^^]^.

Capnography has been regarded as a potentially useful monitoring method in evaluating the effectiveness of CPR maneuvers, despite limitations and controversies surrounding the subject^[^^15^^]^. Animal and human studies have shown a good correlation between PetCO_2_ and DC during stages of decreased blood flow and during CPR^[^^16^^]^. PetCO_2_ can reflect the pulmonary blood flow generated in CPR if CO_2_ production and alveolar ventilation are relatively constant during resuscitation maneuvers; however, it is difficult to be measured when CPR is stopped because of changes in the alveolar dead space and minute volume ratio, which affects the correlation between PetCO_2_ and DC^[^^17^^]^.

Some studies suggest that the increase in PetCO_2_ during CA is a predictor of the success of the CPR. Considering the event of cardiac arrest, CPR and the post-event, we can find several changes in PetCO_2_ levels. It is known that the values of PetCO_2_ at the beginning of ventricular fibrillation (VF) fall significantly, and this reduction is attributed to decreased pulmonary blood flow, which is insufficient to carry and eliminate the CO_2_ produced in the tissues. Evidently, in extreme cases of low DC, there would also be a lower CO_2_ production because of the anaerobic metabolism, given the low O_2_ supply to the tissues. Once CPR is started, and it is effective in oxygenating the blood and increasing the tissue flow, PetCO_2_ values also increase, as an increase in blood flow must occur in the pulmonary capillaries, which in turn results in the exhalation of CO_2_. When the return of spontaneous circulation (ROSC) occurs, those values increase significantly, reaching levels comparable to those before CA. These changes are useful to quantify the effectiveness and success of the CPR maneuvers as well as to assess the cardiorespiratory status of the patient after ROSC. It has been observed that there is no survival for a PetCO_2_ < 5 mmHg^[^^18^^]^.

Some studies have mentioned a few factors that can affect the levels of CO_2_ eliminated by expiration. Among them, we can mention alveolar ventilation, DC, the area of distribution of blood flow in the body, and the production of CO_2_ by tissues. Some authors also reported that the measurement of CO_2_ is not able to reflect the certain success of CPR, since the results do not confirm those reported in studies that have measured other parameters^[^^19^^]^. However, capnography is still used and regarded as the best and most effective non-invasive method of measuring the elimination of CO_2_ in Emergency Rooms, Surgery Centers, and ICU in cases of CA, being considered essential when performing CPR, for decision-making, assessment of its initial success (ROSC), and subsequent clinical evolution (cardiorespiratory stabilization).

The objective of this study was to assess if PetCO_2_, VCO_2_, and coronary perfusion pressure (CorPP) values are useful in predicting the success of ROSC in an animal model of CA/CPR using vasopressor agents.

## METHODS

This study was approved by the Institutional Review Committee for Experiments with Animals (EAEC-IB-Unicamp-1276-1/2007) and it was conducted in the laboratory of Experimental Surgery and Medicine, School of Medical Sciences - Universidade Estadual de Campinas (UNICAMP), São Paulo, Brazil.

The methods used were the same as the ones described in the novel article of Ovalle et al.^[^^20^^]^, using forty-two Large-White, immature swine, weighing approximately 20 kg, which presented ROSC. Under anesthesia with ketamine (10 mg.kg^-1^ intramuscularly) and thiopental (25 mg.kg^-1^ intravenously), the animals were intubated endotracheally and ventilated with FiO_2_=0.21 (and positive pressure at the end of exhalation of 0 cmH2O), a fixed respiratory rate (10 cpm), and a tidal volume ranging from 15 to 20 mL/kg (Ventilator DX-3010^®^, Dixtal, Brazil), in order to maintain a PetCO_2_ between 36-44 mmHg (Respiratory Profile Monitor CO_2_SMO Plus 8100^®^, Dixtal/Novametrix, Respironics, Murrisville, PA, USA). Surgical vascular catheterizations were performed to measure pressure in the thoracic aorta and the right atrium (DX-2020^®^, Dixtal, Brazil).

Using a bipolar pacemaker placed on the right ventricular cavity, we induced VF, which remained without assistance for 10 minutes. Then, the animals were kept in the supine position and reattached to the mechanical ventilator, and we started CPR (100 compressions/10 ventilations/min, continuously, without alternating with chest compressions).

After two minutes, the animals were allocated into four groups (randomized and blind), receiving via central IV: Group 1 - Adrenaline (ADR - 45 µg.kg^-1^); Group 2 - saline-placebo (10 ml); Group 3 - Terlipressin (TP* - *Glypressin^®^, Laboratórios Ferring Ltda., Brazil - 20 µg.kg^-1^); and Group 4 - TP (20 µg.kg^-1^) + ADR (45 µg.kg^-1^). All drugs were diluted in saline solution (10 ml), in equal syringes, thus the main resuscitator did not know what drug was being administered.

Two minutes after injecting the drugs, defibrillation was performed with sequential shocks (every 15 seconds) of 200 J (Biphasic Defibrillator, Cardiomax, Instramed, Brazil), until ROSC, a pace other than VF was obtained or 2 minutes of attempts had elapsed. We set the return of spontaneous circulation as the recovery of spontaneous heart rate with a systolic blood pressure ≥ 60 mmHg for ≥ 5 minutes. The animals were considered as survivors when they remained alive, with a systolic blood pressure ≥ 60 mmHg, without the use of additional vasopressor agents for 30 minutes after ROSC.

During spontaneous circulation, CorPP was calculated as the difference between mean arterial pressure and mean central venous pressure. During CPR maneuvers, CorPP was calculated as diastolic arterial pressure (decompression) minus central venous pressure (decompression)^[^^21^^]^. At the completion of the experiment, all animals resurrected were killed with an overdose of thiopental and 19.1% potassium chloride.

### Statistical Analysis

Initially, we performed a descriptive analysis, presented in the form of tables with frequency and measures of the location and dispersion of values. For comparison of the parameters assessed in only one moment between the groups, we used the Kruskal-Wallis test. For comparison of the parameters measured among the groups and times, we used the analysis of variance (ANOVA) for repeated measures, with transformation by posts, followed by multiple comparisons through the Tukey test for the location of differences between groups and the profile test for contrasts for the location of the differences between times. To verify the difference between proportions, we used Fisher's exact test. Statistical tests were bilateral and the significance level adopted was 5% (*P*<0.05).

## RESULTS

In Table 1, we describe the values of the following variables:

**Table 1:** Descriptive analysis and comparisons of the variables assessed at baseline (0), 2 min of CPR (2), and 4 min of CPR (4) within and among groups.

	**ADR + TP (n=11)**	**ADR (n=10)**	**Placebo (n=10)**	**TP (n=11)**	***P***
PetCO_2_ 0*	42.5±5.2	39.4+3.5	43.4+3.5	40.4+6	*
VCO_2_ 0¥	121.2+20.5	133.4+53.7	110.2+21.2	119.3+40.8	¥
CorPP 0§	88.9±21	89.7+19.5	91.0+18.3	85.5+23.2	§
PetCO_2_ 2*	50.7+13.1	37.4+14	43.4+16.3	44.2+17.7	*
VCO_2_ 2¥	48.7±27.5	57.5+30.2	47.5+21.2	45.3+24.5	¥
CorPP 2§	21.3+10.4	12.6+12.1	22.7+13.5	20.2+15.5	§
PetCO_2_ 4*	44.3+15	43.6+19.3	46.3+12.4	51.0+19.4	*
VCO_2_ 4¥	46.4+31.1	49.6+32.9	49.2+21.3	35.5+25.5	¥
CorPP 4§	44.6+13.1	53.1+15.2	13.7+12	7.0+10.5	§

ADR=adrenaline; TP=terlipressin; PetCO_2_=end-tidal carbon dioxide tension (mmHg); VCO_2_=carbon dioxide excretion; CorPP=coronary perfusion pressure

* Results of the ANOVA for repeated measurements with transformation for posts: Effect of group, P=0.5427; effect of time, P=0.1317; group/time interaction, P=0.3043.

¥ Results of the ANOVA for repeated measurements with transformation for posts: Effect of group, P=0.7530; effect of time, P=<0.0001; group/time interaction, P=0.8622. Differences between times (profile test for contrast): Basal' 2'CPR, *P*<0.0001; basal' 4'CPR, *P*<0.0001; 2'CPR' 4'CPR, P=0.2286.

§ Results of the ANOVA for repeated measurements with transformation for posts: Effect of group, P=0.0012; effect of time, *P*<0.0001; group/time interaction, *P*<0.0001. Fixed group and time comparison (profile test for contrast): Placebo group, *P*<0.0001; Basal ↔ 2'CPR, *P*<0.0001; basal ↔ 4'CPR, *P*<0.0001; 2'CPR ↔ 4'CPR, P=0.06. Adrenaline group, *P*<0.0001; Basal ↔ 2'CPR, *P*<0.0001; Basal ↔ 4'CPR, *P*<0.0014; 2'CPR ↔ 4'CPR, *P*<0.0001. Terlipressin group, *P*<0.0001; Basal ↔ 2'CPR, *P*<0.0001; Basal ↔ 4'CPR, *P*<0.0001; 2'CPR ↔ 4'CPR, P=0.0072. ADR+TP group, *P*<0.0001; Basal ↔ 2'CPR, *P*<0.0001; Basal ↔ 4'CPR, P=0.0003; 2'CPR ↔ 4'CPR, *P*<0.0001. Setting time and comparing groups (Tukey test): Basal, without differences, P=0.9369; 2'CPR, without differences, P=0.2341; 4'CPR, with differences in Placebo and Adrenaline; Adrenaline and Terlipressin; ADR+TP and Placebo; Terlipressin and ADR+TP, *P*<0.0001.

- PetCO_2_ (mmHg) measured at baseline (before induction of CA), two minutes of CPR (before the injection of drugs), and 4 min of CPR (2 min after the injection of drugs and immediately before ventricular defibrillation); there were no statistically significant differences between the groups;
- VCO_2_ (mL/min) measured at baseline (before induction of CA), two minutes of CPR (before the injection of drugs), and 4 min of CPR (2 min after the injection of drugs and immediately before ventricular defibrillation); although there was a statistically significant decrease in VCO_2_ at 2 min and 4 min of CPR in relation to baseline, there were no statistically significant differences between the groups at any moment;
- CorPP (mmHg) measured at baseline (before induction of CA), two minutes of CPR (before the injection of drugs), and 4 min of CPR (2 min after the injection of drugs and immediately before ventricular defibrillation). A statistically significant increase in CorPP between the 2 min of CPR (before the injection of drugs) and 4 min of CPR (2 min after the injection of drugs) was observed in the ADR and ADR+TP groups compared to the placebo and isolated TP groups (which was equal to placebo);
- Baseline rectal temperature (ºC): ADR: 39.0±0.7; Placebo: 39±0.5; TP: 39.3±0.6; and ADR+TP: 39.6±0.4 (*P*=0.2085).

**Fig. 1:**
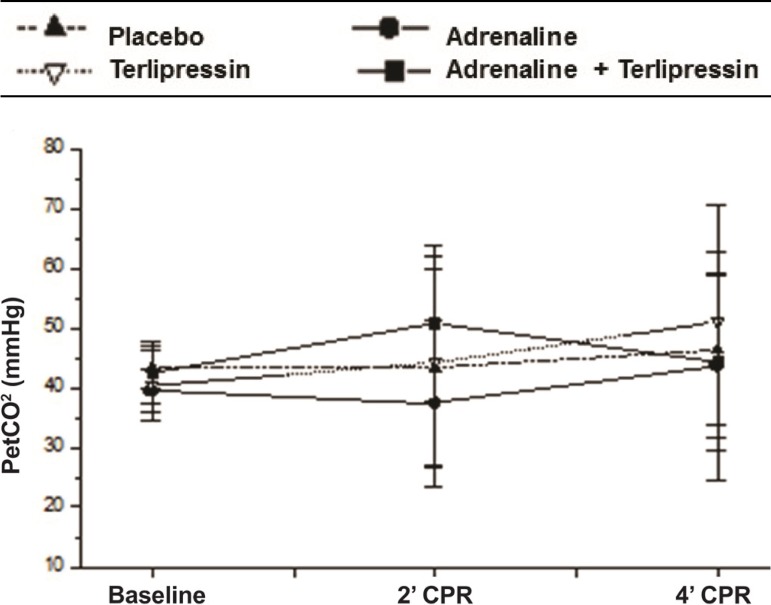
Comparative evolution of the variables assessed at baseline (0), 2 min of CPR (2) and 4 min of CPR in the groups studied. Average value and standard deviation of PetCO_2_ in each moment and group.

**Fig. 2:**
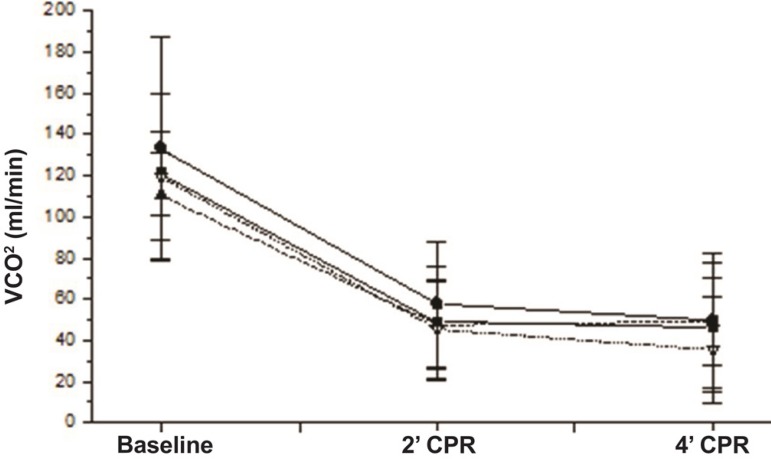
Comparative evolution of the variables assessed at baseline (0), 2 min of CPR (2) and 4 min of CPR in the groups studied. Average value and standard deviation of VCO_2_ in each moment and group.

**Fig. 3:**
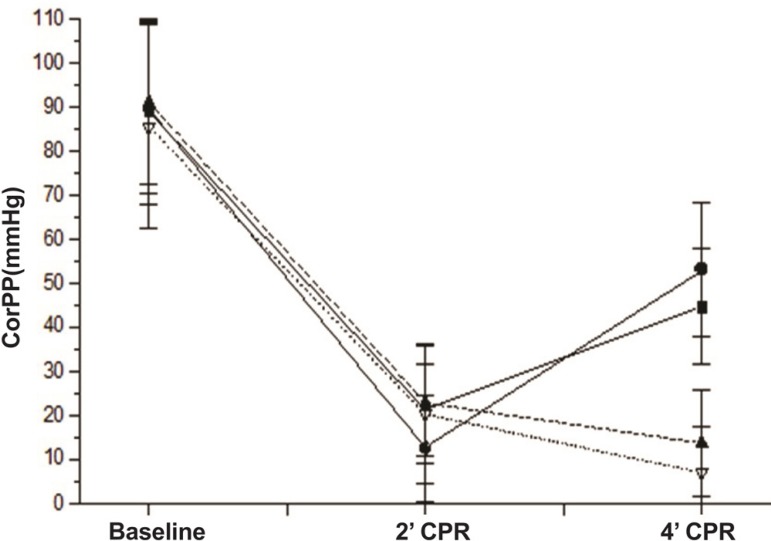
Comparative evolution of the variables assessed at baseline (0), 2 min of CPR (2) and 4 min of CPR in the groups studied. Average value and standard deviation of CorPP in each moment and group.

Those same variables are shown in Figures 1, 2 and 3.

## DISCUSSION

Several studies, both clinical and trial, have investigated the prognostic value of capnography in CA/CPR. The PetCO_2_ measured by capnography in intubated patients has been correlated with the quality of CPR maneuvers and with ROSC, considering that it is directly related to DC, which in turn is directly related to pulmonary blood flow, PetCO_2_ being its reflection^[^^22^^]^. However, no experimental model under invasive mechanical ventilation (IMV), with PEEP=0 cmH_2_O and FiO_2_=0.21, has assessed PetCO_2_, VCO_2_, and CorPP in the prediction of success of ROSC on unassisted CPR for 10 min using terlipressin (a pro-drug, a vasopressin synthetic analogue, with a longer half-life than vasopressin), adrenaline and their combination as vasopressor agents, in addition to placebo.

**Table 2:** Descriptive analysis and comparison of the percentage of survivors among the groups.

**Surviving groups**	**ADR+TP (n=11)**	**ADR (n=10)**	**Placebo (n=10)**	**TP (n=11)**	**Total (n=42)**
No n (%)	2 (18)	2 (20)	8 (80)	10 (91)	22 (52)
Yes n (%)	9 (82)	8 (80)	2 (20)	1 (9)	20 (48)

*P*<0.0001 (Fisher's test)

In several published studies, PetCO_2_ has proved to be a useful variable in assessing the effectiveness of CPR maneuvers and results in different models of CA.

In the 1980s, Sanders et al.^[^^23^^]^ monitored the elimination of CO_2_ (VCO_2_) in experimental studies of CA/CPR. The elimination of CO_2_ was assessed in different types of chest compression, and the study reported that all types of maneuvers increased PetCO_2_. Little more than a decade later, Blumenthal et al.^[^^24^^]^, also in an experimental study, measured PetCO_2_ and VCO_2_ and concluded that higher values of PetCO_2_ during and after CPR were associated with a better prognosis.

In our study, there was no statistically significant association between the times and the registered values for PetCO_2_ with ROSC, although a sudden and immediate increase in PetCO_2_ at the beginning of the CPR was observed, which expresses the pulmonary blood flow, and thus, DC. The data indicates that PetCO_2_, both at 2 min and 4 min of CPR (more importantly, because it was after the administration of the drugs), is not correlated with ROSC rates, *i.e*., it was not different among the four groups. Nevertheless, we have to consider that in our study the CA/CPR model was performed on very young (immature) animals, being an extreme CA model, for 10 min without any assistance, and, after this period, the animals were ventilated with an initial FiO_2_ (0.21). We should also highlight the fact that we did not alternate between ventilation and compression, which may have compromised the effectiveness of the alveolar ventilation.

In relation to VCO_2_ at 2 and 4 min of CPR, it did not differ statistically between the four groups and, therefore, it was not indicative of ROSC. On the other hand, CorPP proved to be significantly higher in the ADR and ADR+TP groups compared to the Placebo and Terlipressin groups at 4 minutes of CPR, which indicates that CorPP was a predictive factor for ROSC.

Human studies use other variables to assess the successful prediction of ROSC. Different criteria are used, but capnography is also the main focus of the prognostic assessment after CA. One of the first studies in the 1980s by Garnett et al.^[^^25^^]^ assessed patients under IMV who presented CA, and they concluded that the monitoring of PetCO_2_ during CPR can be useful and serve as a guide during resuscitation maneuvers.

Another clinical study, conducted by Sanders et al.^[^^26^^]^, assessed PetCO_2_ in patients subjected to CPR. The authors reported that all patients listed and those who presented ROSC showed an average PetCO_2_ ≥ 10 mmHg. None of the patients with an average PetCO_2_ ≤ 10 mmHg presented ROSC. The data in this prospective clinical test indicate that the monitoring of PetCO_2_ during CPR can be useful in the prediction of ROSC in CPR in humans.

PetCO_2_ values have been correlated with CorPP and ROSC rate. Thus, in an animal model (dogs) of CA/CPR, Sanders et al.^[^^27^^]^, in another study, found a significant correlation (*P*<0.01) between PetCO_2_ and CorPP. The data in this study were confirmed by the same group, in the same year, with small variations in the method. However, the actual physiological relationship between CorPP and PetCO_2_ during CPR remains uncertain^[^^23^^]^.

In our study, we have observed a significant increase in CorPP (*P*<0.0001) in the 4^th^ minute of CPR (after drugs) in relation to the 2^nd^ min (before drugs) of CPR in the ADR and ADR+TP groups. However, the same behavior was no observed in PetCO_2_ and VCO_2_.

The use of vasopressor agents is suggested in the first cycles of CPR^[^^25^^]^. For different types of CA, vasopressor, adrenaline or vasopressin can be administered in order to increase myocardial and cerebral blood flow. In the study of Ovalle et al.^[^^20^^]^, the use of some vasopressor agents during CPR was assessed. It was observed that ADR and its combination with terlipressin, but not isolated terlipressin, were effective in increasing CorPP and ROSC. Furthermore, the ADR+TP combination provided greater hemodynamic stability after ROSC in the surviving animals, suggesting that TP can be a useful drug in handling hypotension after CPR^[^^28^^]^.

In most published studies, both clinical and experimental ones, the initial, final, maximum and minimum readings show higher values of PetCO_2_ in patients who presented ROSC. These authors highlight that clinical studies receive influences from diseases already present in patients, and this factor should be considered^[^^18^^]^.

The PetCO_2_ values assessed in many (clinical and experimental) studies can assist in verifying the effectiveness of CPR maneuvers in order to guide, with due caution, the actual results of resuscitation, since very low PetCO_2_ values may indicate that there is no more reason to continue the efforts of CPR^[^^29^^]^.

In short, PetCO_2_ and VCO_2_ values obtained by volumetric capnography have differed from the findings in some published studies. In addition, those values are not correlated with hemodynamic variables (CorPP) or with ROSC rates in our experimental model with immature swine, in which CA was induced by ventricular fibrillation and the animals remained without assistance for 10 min with subsequent CPR. It should be noted that the animals were under IMV, with FiO_2_=0.21 and PEEP=0 cmH_2_O, and we used vasopressor agents (ADR and TP; isolated or in association).

## CONCLUSION

From the results obtained in the study, both PetCO_2_ and VCO_2_ showed no correlation with ROSC, although VCO_2_ was 50% lower during the CPR maneuvers (would it be more sensitive in the detection of a decrease in pulmonary blood flow?). Although we cannot affirm with certainty, some hypotheses were made to explain this fact, namely: FiO_2_=0.21 during the entire experiment, immaturity of the animals, time of non-assistance after CA (10 minutes), and positive pressure (IMV) in the CPR, which may have led to an even sharper decrease in the right preload. Thus, further studies are needed to verify the value of volumetric capnography (PetCO_2_ and VCO_2_) to guide the effectiveness of CPR maneuvers, especially with the use of adjuvant vasopressor agents.

**Table:** 

**Authors' roles & responsibilities**	
ACLM	Analysis and/or data interpretation; manuscript writing or critical review of its content; final manuscript approval
LCM	Analysis and/or data interpretation; manuscript writing or critical review of its content; final manuscript approval
IAP	Analysis and/or data interpretation; manuscript writing or critical review of its content; final manuscript approval;
CCISO	Conception and design study; realization of operations and/ or trials; analysis and/or data interpretation; manuscript writing or critical review of its content
SA	Conception and study design; execution of operations and/ or trials; analysis and/or data interpretation; manuscript writing or critical review of its content; final manuscript approval;
MMM	Execution of operations and/or trials; analysis and/or data interpretation; manuscript writing or critical review of its content; final manuscript approval

## References

[1] Gravenstein JS, Jaffe M, Paulus DA (2011). Capnography:clinical aspects. Carbon dioxide over time and
volume.

[2] Toneloto MG, Moreira MM, Bustorff-Silva JM, Souza GF, Martins LC, Dragosavac D (2015). Adjustable inspiratory occlusion valve in experimental
bronchopleural fistula. A new therapeutic perspective. Acta Cir Bras.

[3] Antonelli RQ, Moreira MM, Martins LC, Negro MS, Baldasso TA, Tincani AJ (2015). Evaluation of the efficiency of the atraumatic endotracheal tube
in the pulmonary-gas exchange: an experimental study. Braz J Cardiovasc Surg.

[4] Oliveira DG, Toneloto MG, Moreira MM, Bustorff-Silva JM, Souza GF, Martins LC (2015). Hemodynamic, ventilatory and gasometric evaluation of an
experimental bronchopleural fistula. Acta Cir Bras.

[5] Pereira DJ, Moreira MM, Paschoal IA, Martins LC, Metze K, Moreno H (2011). Near-fatal pulmonary embolism in an experimental model:
hemodynamic, gasometric and capnographic variables. Rev Bras Cir Cardiovasc.

[6] Ferreira JH, Terzi RG, Paschoal IA, Silva WA, Moraes AC, Moreira MM (2006). Mechanisms underlying gas exchange alterations in an experimental
model of pulmonary embolism. Braz J Med Biol Res.

[7] Silva SM, Paschoal IA, De Capitani EM, Moreira MM, Palhares LC, Pereira MC (2016). COPD phenotypes on computed tomography and its correlation with
selected lung function variables in severe patients. Int J Chron Obstruct Pulmon Dis.

[8] Oliveira PM, Moreira MM (2015). Capnography: a feasible tool in clinical and experimental
settings. Respir Care.

[9] Veronez L, Pereira MC, Silva SM, Barcaui LA, De Capitani EM, Moreira MM (2014). Volumetric capnography for the evaluation of chronic airways
diseases. Int J Chron Obstruct Pulmon Dis.

[10] Ribeiro MA, Silva MT, Ribeiro JD, Moreira MM, Almeida CC, Almeida-Junior AA (2012). Volumetric capnography as a tool to detect early peripheral lung
obstruction in cystic fibrosis patients. J Pediatr (Rio J).

[11] Veronez L, Moreira MM, Soares ST, Pereira MC, Ribeiro MA, Ribeiro JD (2010). Volumetric capnography for the evaluation of pulmonary disease in
adult patients with cystic fibrosis and noncystic fibrosis
bronchiectasis. Lung.

[12] Moreira MM, Terzi RG, Paschoal IA, Martins LC, Oliveira EP, Falcão AL (2010). Thrombolysis in massive pulmonary embolism based on the
volumetric capnography. Arq Bras Cardiol.

[13] Welch A (2003). Capnography, mainstream and sidestream modules.

[14] Blumenthal SR, Voorhess WD (1997). The relationship between airway carbon dioxide excretion and
cardiac output during cardiopulmonary resuscitation. Resuscitation.

[15] Kalenda Z (1978). The capnogram as a guide to the efficacy of cardiac
massage. Resuscitation.

[16] Moreira MM, Terzi RGG, Ferreira ELA, Moraes AC, Silva WA (2005). Correlação entre a pressão
expiratória final de CO2 e o débito cardíaco no choque
hemorrágico experimental. RBTI.

[17] Sehra R, Underwood K, Checchia P (2003). End tidal CO2 is a quantitative measure of cardiac
arrest. Pacing Clin Electrophysiol.

[18] Blumenthal SR, Voorhess WD (1997). The relationship between airway carbon dioxide excretion and
cardiac output during cardiopulmonary resuscitation. Resuscitation.

[19] Asplin BR, White RD (1995). Prognostic value of end-tidal carbon dioxide pressures during
out-of-hospital cardiac arrest. Ann Emerg Med.

[20] Ovalle CCIS, Moreira MM, Martins LC, Araujo S (2011). A eficácia da terlipressina versus adrenalina na
ressuscitação cardiopulmonar em suínos. Rev Bras Anestesiol.

[21] Paradis NA, Martin GB, Rivers EP, Goetting MG, Appleton TJ, Feingold M (1990). Coronary perfusion pressure and the return of spontaneous
circulation in human cardiopulmonary resuscitation. JAMA.

[22] Gudipati CV, Weil MH, Bisera J, Deshmukh HG, Rackow EC (1988). Expired carbon dioxide: a noninvasive monitor of cardiopulmonary
resuscitation. Circulation.

[23] Sanders AB, Ewy GA, Bragg S, Atlas M, Kern KB (1985). Expired PCO2 as a prognostic indicator of successful
resuscitation from cardiac arrest. Ann Emerg Med.

[24] Blumenthal SR, Voorhees WD (1997). The relationship of carbon dioxide excretion during
cardiopulmonary resuscitation to regional blood flow and
survival. Resuscitation.

[25] Garnett AR, Ornato JP, Gonzalez ER, Johnson EB (1987). End-tidal carbon dioxide monitoring during cardiopulmonary
resuscitation. JAMA.

[26] Sanders AB, Kern KB, Otto CW, Milander MM, Ewy GA (1989). End-tidal carbon dioxide monitoring during cardiopulmonary
resuscitation. A prognostic indicator for survival. JAMA.

[27] Sanders AB, Atlas M, Ewy GA, Kern KB, Bragg S (1985). Expired PCO2 as an index of coronary perfusion
pressure. Am J Emerg Med.

[28] Gonzalez MM, Timerman S, Gianotto-Oliveira R, Polastri TF, Canesin MF, Schimidt A, Sociedade Brasileira de Cardiologia (2013). First guidelines of the Brazilian Society of Cardiology on
Cardiopulmonary Resuscitation and Cardiovascular Emergency
Care. Arq Bras Cardiol.

[29] Nordseth T, Olasveengen TM, Kvaloy JT, Wik L, Steen PA, Skogvoll E (2012). Dynamic effects of adrenaline (epinephrine) in out-of-hospital
cardiac arrest with initial pulseless electrical activity
(PEA). Resuscitation.

